# Effects of Leucine Supplementation and Serum Withdrawal on Branched-Chain Amino Acid Pathway Gene and Protein Expression in Mouse Adipocytes

**DOI:** 10.1371/journal.pone.0102615

**Published:** 2014-07-22

**Authors:** Abderrazak Kitsy, Skyla Carney, Juan C. Vivar, Megan S. Knight, Mildred A. Pointer, Judith K. Gwathmey, Sujoy Ghosh

**Affiliations:** 1 Division of Cardiometabolic Disorders, Biomedical Biotechnology Research Institute, North Carolina Central University, Durham, North Carolina, United States of America; 2 Boston University School of Medicine, Boston, Massachusetts, United States of America; 3 Program in Cardiovascular and Metabolic Disorders, Duke-NUS Graduate Medical School, Singapore, Singapore; University of Minnesota - Twin Cities, United States of America

## Abstract

The essential branched-chain amino acids (BCAA), leucine, valine and isoleucine, are traditionally associated with skeletal muscle growth and maintenance, energy production, and generation of neurotransmitter and gluconeogenic precursors. Recent evidence from human and animal model studies has established an additional link between BCAA levels and obesity. However, details of the mechanism of regulation of BCAA metabolism during adipogenesis are largely unknown. We interrogated whether the expression of genes and proteins involved in BCAA metabolism are sensitive to the adipocyte differentiation process, and responsive to nutrient stress from starvation or BCAA excess. Murine 3T3-L1 preadipocytes were differentiated to adipocytes under control conditions and under conditions of L-leucine supplementation or serum withdrawal. RNA and proteins were isolated at days 0, 4 and 10 of differentiation to represent pre-differentiation, early differentiation and late differentiation stages. Expression of 16 BCAA metabolism genes was quantified by quantitative real-time PCR. Expression of the protein levels of branched-chain amino acid transaminase 2 (Bcat2) and branched-chain alpha keto acid dehydrogenase (Bckdha) was quantified by immunoblotting. Under control conditions, all genes displayed induction of gene expression during early adipogenesis (Day 4) compared to Day 0. Leucine supplementation resulted in an induction of *Bcat2* and *Bckdha* genes during early and late differentiation. Western blot analysis demonstrated condition-specific concordance between gene and protein expression. Serum withdrawal resulted in undetectable Bcat2 and Bckdha protein levels at all timepoints. These results demonstrate that the expression of genes related to BCAA metabolism are regulated during adipocyte differentiation and influenced by nutrient levels. These results provide additional insights on how BCAA metabolism is associated with adipose tissue function and extends our understanding of the transcriptomic response of this pathway to variations in nutrient availability.

## Introduction

Branched-chain amino acids (BCAAs) make up approximately 40% of the free essential amino acids in blood and play important roles in skeletal muscle growth and maintenance, primarily as protein synthesis substrates [1]. The most widely studied BCAA, L-leucine, has also been shown to play additional roles in skeletal muscle [2], including the regulation of translation initiation [3], modulation of insulin/PI3-kinase signaling [4], [5], provision of metabolic fuel [6], and donation of nitrogen for alanine and glutamine synthesis [7]. Leucine has also been implicated in muscle insulin resistance (via inhibitory phosphorylation of the insulin receptor substrate-1 by mTOR kinase) [8], [9], although this finding has not been fully supported by other studies [10]. Several studies have also investigated the potentially beneficial role of BCAAs in sparing lean body mass during weight loss due to caloric restriction and muscle wastage with aging [2], [11].

While the majority of studies on BCAAs have focused on their roles in skeletal muscle, obesity-associated elevations in post-absorptive plasma levels of BCAAs had been observed in some early studies [12], [13], if not in all of them [14], [15]. An example of earlier evidence pointing to a role of adipose tissue in obesity-associated BCAA dysregulation comes from observations of reduced expression of mitochondrial branched-chain amino acid aminotransferase (*Bcat2*) and branched-chain α-keto acid dehydrogenase (*Bckdha*) enzymes in epididymal fat from ob/ob mice and Zucker rats, compared to their lean counterparts [16]. Using gene expression pathway analysis, a more recent study also identified the adipose tissue BCAA metabolic pathway as the most significantly altered pathway in mice carrying an adipose-tissue specific knockout of the glucose transporter, Glut4 [17]. In humans, a comprehensive metabolomic profiling study [18] revealed a BCAA-related metabolite signature differentiating obese from lean human subjects. The BCAA-related signature also displayed a significant linear relationship to insulin resistance [19], correlated with HbA1c levels in weight-matched type 2 diabetic vs. non-diabetic women [20], and predicted improvements in insulin sensitivity during weight loss [21]. The association of circulating BCAA and insulin action was further found to be modified by body mass index (BMI), resting respiratory quotient, presence of type 2 diabetes, and gender [22]. A study investigating obesity-discordant monozygotic twins demonstrated significant downregulation of adipose tissue BCAA metabolism gene expression, and a negative correlation with insulin sensitivity in the obese twin [23]. In our own studies, a significant downregulation of adipose tissue BCAA metabolism gene expression was also observed in overweight, hypertriglyceridemic subjects with adipose-tissue insulin-resistance, compared to BMI-matched controls [24]. The weight of evidence from these studies suggests an association between circulating BCAA levels and insulin resistance (rather than with obesity *per se*), although evidence for whether elevated BCAA is a causal driver of insulin resistance, or a consequence thereof, is currently equivocal [25].

Nevertheless, it has become increasingly clear that adipose tissue is a significant depot for systemic BCAA homeostasis. This is based on multiple studies involving the measurement of BCAA enzyme activity levels in whole-tissue extracts, kinetics of leucine utilization in white adipose tissue (WAT), and comparative gene expression analysis of branched-chain aminotransferase (Bcat2), and branched-chain keto acid dehydrogenase (Bckdh) enzyme components in WAT [17], [26]–[29]. However, a more comprehensive understanding of the role and significance of adipose tissue BCAA metabolism is still wanting and several important gaps remain. For example, most of these studies to date have been restricted to investigating genetic models of obesity and have focused on comparisons between adipose tissues obtained from obese and lean samples that are heterogeneous both with respect to resident cell types and stages of adipocyte differentiation. Additionally, with few exceptions [28], most studies have largely concentrated on the two common steps in BCAA catabolism, leaving the status of the downstream BCAA-specific genes largely unexamined. Since intermediates of BCAA-specific metabolism feed directly into several other metabolic processes (e.g., citrate cycle, biosynthesis of aromatic amino acids and cholesterol, metabolism of fatty acids, etc.), changes in the expression of genes that regulate BCAA metabolism intermediates are expected to have additional consequences on cellular metabolism. Furthermore, given the potentially novel role of BCAA metabolism in obesity-associated insulin resistance, there is strong impetus to carefully delineate the relationship of BCAA metabolism to adipocyte biology. Whereas where several studies have investigated the primary steps of BCAA catabolism in response to nutrient availability and starvation in liver, heart or skeletal muscle (such as the effects of whole food and protein starvation on Bckdha activity) [30]–[34], the role of nutrient variation on adipocyte BCAA function is relatively unknown. In the current work, we have investigated some aspects of this problem by using 3T3-L1 murine adipocytes as a model system, and interrogated changes in an expanded repertoire of BCAA metabolism gene and protein components during adipocyte differentiation under conditions of serum withdrawal or exposure to physiologically relevant increases in L-leucine. This work extends the recently reported findings from Lackey, et al. who also tested the expression of a focused subset of BCAA-associated genes in differentiating 3T3-L1 adipocytes under conditions of PPAR-gamma agonism [28].

## Materials and Methods

### Materials

The Swiss mouse embryo derived preadipocyte cell line, 3T3-L1, was obtained from Dr. Howard Green (Harvard University). Dulbecco's Modified Eagle's Medium (DMEM), penicillin/streptomycin solution, phosphate-buffered saline (PBS), trypsin-EDTA, fetal bovine serum and calf serum were purchased from Invitrogen (Carlsbad, CA). Biorad Protein Assay reagent and all protein gel electrophoresis and Western blotting apparatus were purchased from Biorad (Hercules, CA). SuperSignal Femto chemiluminescence substrate was purchased from Pierce (Rockford, IL). Pre-stained protein and RNA molecular weight markers, gentamycin solution, agarose (molecular biology grade), and SYBR green PCR Master Mix were obtained from Fisher Scientific (Pittsburgh, PA). L-Leucine (L8912, cell culture grade) was obtained from Sigma Aldrich (St. Louis, MO). Polyclonal antibodies to mouse Bcat2 (ab95976) and Bckdha (ab90691) were obtained from Abcam (Cambridge, MA). Rabbit monoclonal antibody to mouse mitogen activated protein kinase (Mapk) and horseradish peroxidase linked anti-rabbit IgG (secondary antibody) were purchased from Cell Signaling Technologies, Inc. (Danver, MA).

### Cell culture

3T3-L1 preadipocytes were cultured in growth media (89% DMEM high Glucose, 10% bovine calf serum, antibiotic/antimycotic/Pen-strep) for 2–3 days to confluency and then incubated for an additional 48 hours to induce growth arrest (Day 0). Growth arrested cells were treated with differentiation media (89% DMEM F12, 10%Fetal Bovine Serum, 1% antibiotic/antimycotic/Pen-strep, 250 uM 3-isobutyl-1-methylxanthine (IBMX), 500 uM dexamethasone, 1 uM insulin) for 2 days. At the end of 2 days (Day 2), the cells were further treated with maintenance media (89% DMEM F12, 10%Fetal Bovine Serum, 1% Antibiotic/antimycotic Pen-strep) plus 1 uM insulin for the next 4 days. Cells were thereafter continuously treated with maintenance media every 2 days until the end of the experiment at Day 10. Experiments were performed in Petri dishes or 6-well plates at a starting concentration ranging from 2.38×10^5^–2.80×10^5^ cells/ml. Cell treatments were as follows:

#### Control

Cells were cultured in regular growth, differentiation or maintenance medium as described above and harvested at Day 0, Day 4 and Day 10 for protein and RNA extraction.

#### Serum withdrawal

Cells were grown in growth, differentiation and maintenance medium as described above. The media were replaced with media deprived of bovine calf serum, and cells were incubated in this media for 18 hours immediately prior to Day 0, Day 4 and Day 10 of adipocyte differentiation. At the end of the treatment, cells were harvested for protein and RNA extraction.

#### Leucine supplementation

L-Leucine was dissolved in sterile, molecular-biology grade water to a final concentration of 6.56 g/l (50 mM). This stock solution was added to media to a final concentration of 0.5 mM. Since the media already contains 0.8 mM leucine, this treatment represents a 62.5% increase in leucine levels. This level of increase is in the range of elevations previously observed in *in vivo* leucine overfeeding studies [35] or in post-absorptive plasma of obese mice [16]. Cells were cultured in leucine containing media for 18 hours immediately prior to Day 0, Day 4 and Day 10 and then harvested for protein and RNA extraction.

The choice of exposure time was based on prior literature reports of leucine treatment or serum withdrawal of muscle and other cell types, which typically ranged from 12–24 hrs. For all treatments, the course of adipocyte differentiation was monitored by Oil Red O staining of intracytoplasmic lipid accumulation over time [36] and by quantitative polymerase chain reaction (qPCR) analysis of key differentiation related genes. Oil Red O stained cells were photographed through a Zeiss Axiovert 25 microscope attached to a Zeiss Axiocam MRC camera at 40X magnification using a red filter. Non-specific cell death was quantified by measurement of released lactate dehydrogenase activity into cell culture media [37] via absorbance at 490 nm (Cytotox 96 Non-Radioactive Cytotoxicity Assay Kit, Promega, Madison, WI).

### Quantitative PCR and determination of amplification efficiency

Primers for qPCR were designed via the PrimerBlast (National Center for Biotechnology Information), and Primerquest primer design softwares (Integrated DNA Technologies, Coralville, IA). Exon-spanning primers, with total GC content not exceeding 70% and PCR amplicon not exceeding 150 base pairs, were designed whenever possible. Other thermodynamic and design parameters were used at default values provided by the softwares. The sequences of the primers used in the study are provided in ****. qPCR was performed in a total volume of 20 uL using 96-well microwell plates and a Bio-Rad CFX96 Real-Time PCR Detection System. Total RNA was isolated from cells via the RNeasy mini kit (Qiagen). RNA quality was determined via Agilent Bioanalyzer and all RNA samples were found to have 260/280 ratios >2.0 and the RIN values ranged from 9.2–10 (**Table S2**). For qPCR analysis, total RNA was first converted to cDNA (Thermo Scientific Verso). cDNA synthesis reactions were carried out in a total volume of 20 uL consisting of 4 uL of 5X cDNA synthesis buffer, 2 uL dNTP Mix, 1 uL RNA Primer, 1 uL RT Enhancer, 1 uL Verso Enzyme Mix, 1 uL total RNA (100 ng) sample and 10 uL Molecular Grade Water (G-Biosciences). For qPCR, 5 microliters of cDNA, 25 uL iTaq Fast SYBR Green Supermix with Rox (Bio-Rad) and 125 nM of primers were added to each microwell, to a total volume of 20 uL. The plate was centrifuged for 4 min at 2000 rpm at 4°C prior to qPCR. The PCR was run at 95°C for 3 min, followed by 40 cycles at 95°C for 3 sec and 55°C for 30 sec. All PCRs were performed in triplicate.

To evaluate the efficiency of the amplification, a standard curve was constructed by plotting the median quantification cycle value (C_t_, obtained from 3 replicates) versus six 2-fold serial dilutions of the target cDNA template, starting with 100 ng of cDNA. The slope of the standard curve was calculated by linear regression of C_t_ against log_10_(cDNA) and the efficiency of amplification was determined via the Standard Curve Slope to Efficiency Calculator (www.genomics.agilent.com) according to the relation: *Efficiency  = 10^(−1/slope)^ -1*.

### Western blotting and immunodetection

Western blotting was carried out by first transferring proteins from 10% sodium dodecyl sulfate polyacrylamide gels to PVDF membranes at 100 volts for 1 hour. The membrane was then incubated with 10–15 ml of blocking buffer (5% non-fat milk/Tris-buffered saline) at 4°C overnight. Primary antibodies of interest were added to the membrane at dilutions ranging from 1∶1000–1∶10,000, and further incubated at 4°C overnight. Horseradish peroxidase conjugated anti-rabbit secondary antibody was used at a dilution of 1∶2000 for 1 hour. ECL detection was performed with Pierce ECL Western blotting substrate (ThermoScientific, Rockford, IL). Western blots were scanned on a Biorad Molecular Imager Gel Doc XR+ scanner and quantified by ImageJ (imagej.nih.gov).

### Statistical Analysis

Raw data for qPCR was captured via the CFX manager software (Biorad) and converted to cycle numbers corresponding to the threshold of detection for gene expression (Ct). Quantitation of the results was performed via the Comparative C_t_ method [38], using *Gapdh* as an internal calibrator gene. Summary statistics for gene and protein expression data (mean ± standard deviation) and comparison of groups via 2 way analysis of variance (ANOVA) was carried out in JMP statistical software (SAS, Cary, NC). For ANOVA, both main effects (time, treatment) and interaction effects (time * treatment) were considered. Statistical significance was set at the p<0.05 level.

## Results

### Effects of treatments on 3T3-L1 differentiation program

**Figure 1:**
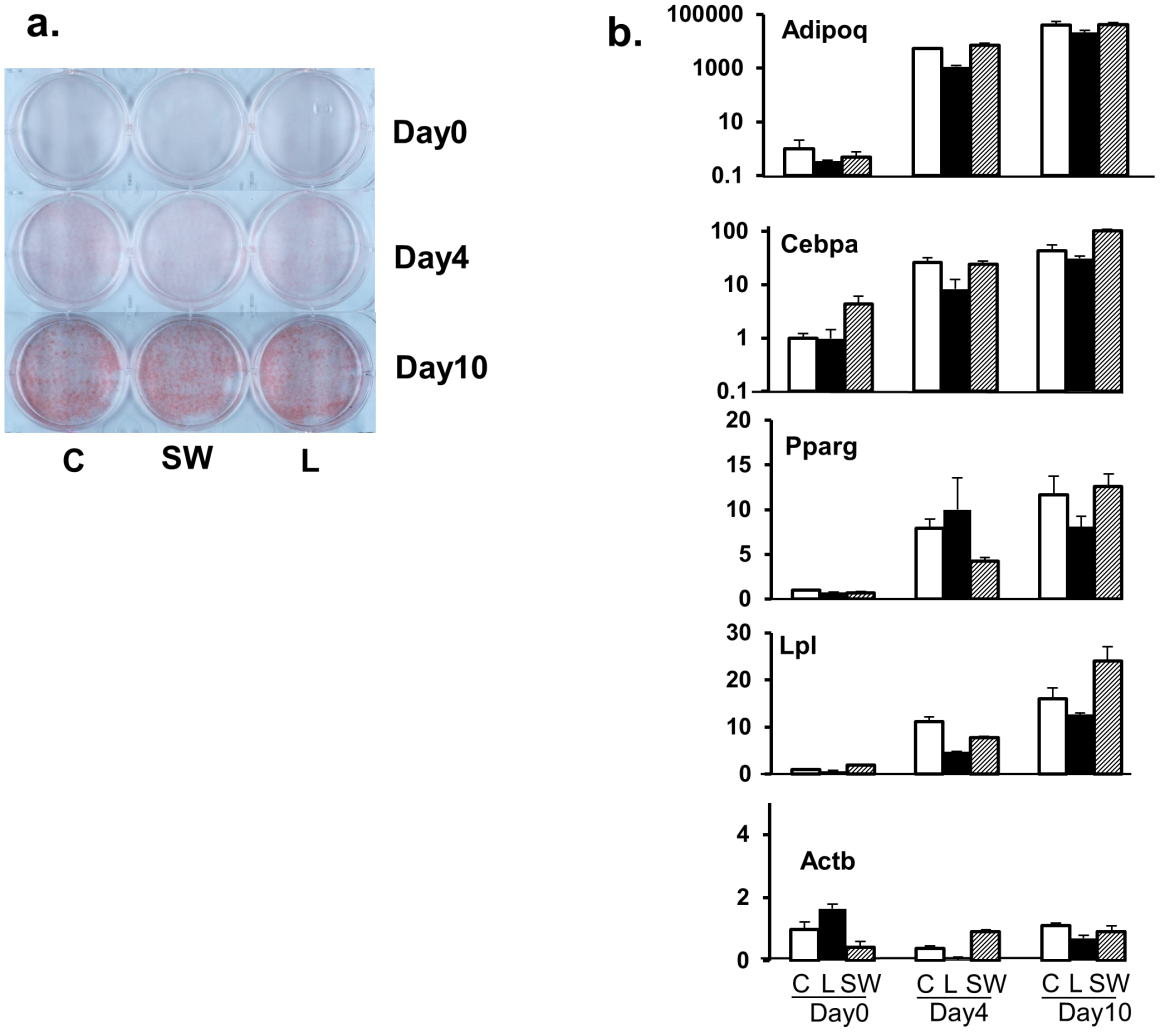
Effects of leucine supplementation and serum withdrawal on 3T3-L1 adipocyte differentiation. **a.** Oil-red O staining of cell cultures at Day 0, Day 4 and Day 10 under *control, leucine or serum withdrawal* conditions. Cells were grown under the defined conditions for the specified periods of time and stained for total neutral lipids. **b.** Quantitative PCR analysis of key genes associated with adipocyte differentiation. Gene names are indicated on the plots. Gene expression abundances were estimated by the ΔΔQ method after normalizing to GAPDH and referencing to Control, Day 0 samples. Due to the relatively high range of fold-change for adiponectin and CEBP-alpha, their relative abundances are expressed in the log_10_ scale. The treatments and stages of differentiation are indicated at the bottom (C,*control*; L, *leucine*; SW, *serum-withdrawal*).

The effects of the different treatments on adipocyte differentiation and adipocyte cell death were monitored separately for each treatment. We did not observe significant treatment-specific effects on 3T3-L1 adipocyte differentiation, as measured by Oil Red O staining and quantitative PCR analysis of key genes related to adipocyte differentiation (**Figure 1**). Compared to *control*, a statistically significant difference in LDH activity was noted in adipocytes exposed to *serum-withdrawal* but not *leucine* treated cells, suggesting greater cell death with serum withdrawal (p<0.05). However the difference in the magnitude of the effect was quite small (5.86% in *serum-withdrawal* vs. 3.09% in *control*, respectively).

### Gene selection for qPCR studies

**Figure 2:**
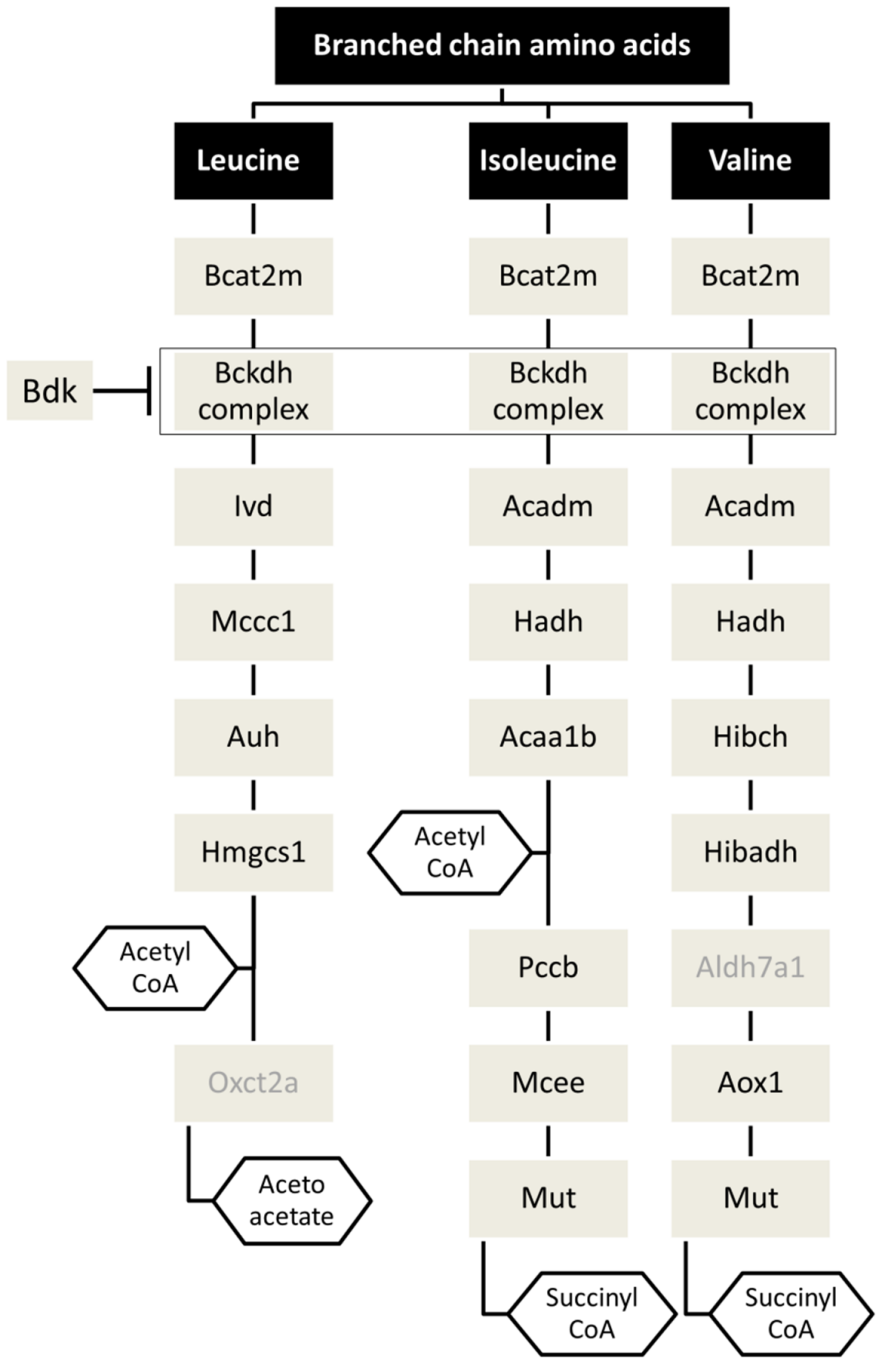
Organization of the BCAA metabolism pathway components studied. Genes involved in Leucine, Valine and Isoleucine metabolism are shown in gray rectangles. End-products of BCAA metabolism are listed as gray hexagons. Genes with grayed out names were not studied further due to unreliable expression levels.

Genes in the BCAA metabolism pathway, acting in the sequential metabolism of leucine, isoleucine and valine to glucogenic and ketogenic precursors (acetoacetate, acetyl CoA and succinyl CoA), were adapted from Herman et al. [17] and are shown schematically in **Figure 2**. A total of 18 BCAA metabolism pathway genes were initially tested in 3T3-L1 preadipocytes and reliable expression was observed for 16 of them (QPCR detection threshold cycles between 17–30; reference Ct for *Gapdh*  = 14.2). The expression levels of the *Aldh7a1* and *Oxct2a* genes were determined to be unreliable (average Ct>35) and were therefore excluded from further analysis (**Figure S1**). Since mRNA quantification estimates are susceptible to bias if the efficiency of PCR amplification differs between the genes being tested [39], we determined the PCR amplification efficiencies of the genes of interest via serial dilutions of the target cDNA template. The amplification efficiencies ranged between 95–110% for all the genes tested (**Table S3**).

### Effects of treatments on BCAA pathway gene expression at different stages of adipocyte differentiation

**Figure 3:**
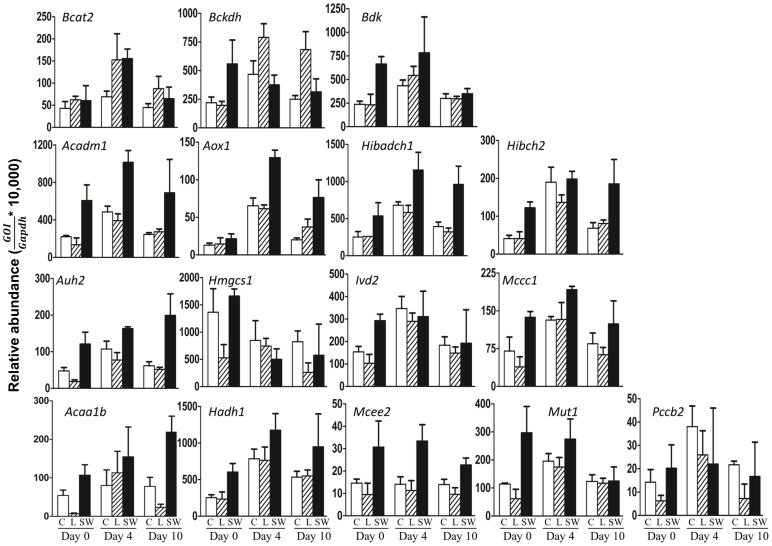
Effect of different treatments on expression levels of BCAA metabolism pathway genes. Quantitative PCR (qPCR) data was generated from three independent cell cultures for each treatment-timepoint combination and analyzed in triplicate. Gene abundances were normalized to *Gapdh* levels and then multiplied by a factor of 10,000 to avoid very small values. The relative abundance measures for each of the replicate cell cultures were combined to provide a summary abundance measure. Panels, from top to bottom – genes common to the metabolism of all 3 BCAAs; genes specific to valine metabolism; genes specific to leucine metabolism; genes specific to isoleucine metabolism. Gene symbols are indicated at the top of each plot. Treatments are shown as C (control, open rectangle), L (leucine, hatched rectangle), and SW (serum withdrawal, black rectangle). Results are shown for each of Day0, Day4 and Day10. *GOI*, gene of interest.

We first interrogated whether any of the 3 treatments changed basal expression levels of the BCAA metabolism genes. We selected 3 timepoints representing pre-differentiation (Day 0), early differentiation (Day 4) and late differentiation (Day 10) of 3T3-L1 adipocytes. Results are shown in **Figure 3**. The highest level of expression was observed for the *Hmgcs1* gene whereas very low expression levels were observed for *Pccb*, and *Mcee*. The expression levels of *Bcat2* were lower compared to *Bckdha* or *Bdk*. Moderate expression was noted for the remaining genes. Generally, the message levels of all genes were increased in the presence of *serum-withdrawal* (with the exception of *Bckdha and Hmgcs1*) compared to *control* or *leucine* supplementation, and for several genes remained higher throughout the entire time course of the experiment (e.g. *Acaa1b*, *Acadm*, *Auh*, and *Hadh*).

The results obtained in Figure 3 were then re-analyzed to determine the effects of treatment on relative changes in gene expression during the course of adipocyte differentiation. We estimated expression fold-changes based on the average expression values at each timepoint with respect to Day 0 which was set at 1.0 (**Figure S2**). In *control* and *leucine* treated samples, we observed a coordinate upregulation of a majority of the tested genes at Day 4, compared to Day 0 or Day 10. However, the expression sensitivity of *Bcat2* and *Bckdha*, the primary regulators of BCAA metabolism, differed from the rest of the genes. In differentiating cells, both *Bcat2* and *Bckdha* mRNA expression were sensitive to leucine manipulation at both days 4 and 10 but not Day 0(fold-change between 2.5–3 fold). The fold-change pattern with serum withdrawal was more variable. For several genes, there was a monotonic increase in fold-change between Day 0 and Day 10 (e.g. *Bdk*, *Auh*, *Pccb*) whereas the opposite was observed for a subset of other genes (*Ivd*, *Hmgcs1*, *Mcee*, *Mut*). Serum withdrawal increased *Bcat2* levels at Day 4 (2.5-fold compared to control) and returned to control levels at Day 10. *Bckdha* levels did not change appreciably under this treatment.

To determine whether any of the observed expression changes were statistically significant, we conducted analysis of variance (ANOVA) on gene expression measures across adipocyte differentiation (three levels) and treatments (three levels). ANOVA identified statistically significant (p<0.05) changes in the expression for several BCAA metabolizing genes. For the majority of the genes, statistical significance was observed for both main effects (time and treatment), whereas interaction effects were observed for a subset of the genes. For example, a two-way analysis of variance on the *Bcat2* gene yielded a main effect for treatment (p<.005), such that the average expression was significantly higher for *leucine* than for *control* or *serum withdrawal*. The main effect of time was also significant, (p<0.0001). However, the interaction effect was not significant, (p<0.18), suggesting that the response to time was not modulated by the individual treatments. Detailed results from the ANOVA analysis are provided in **Table S4**.

### Effects of treatments on key BCAA metabolism protein expression

**Figure 4:**
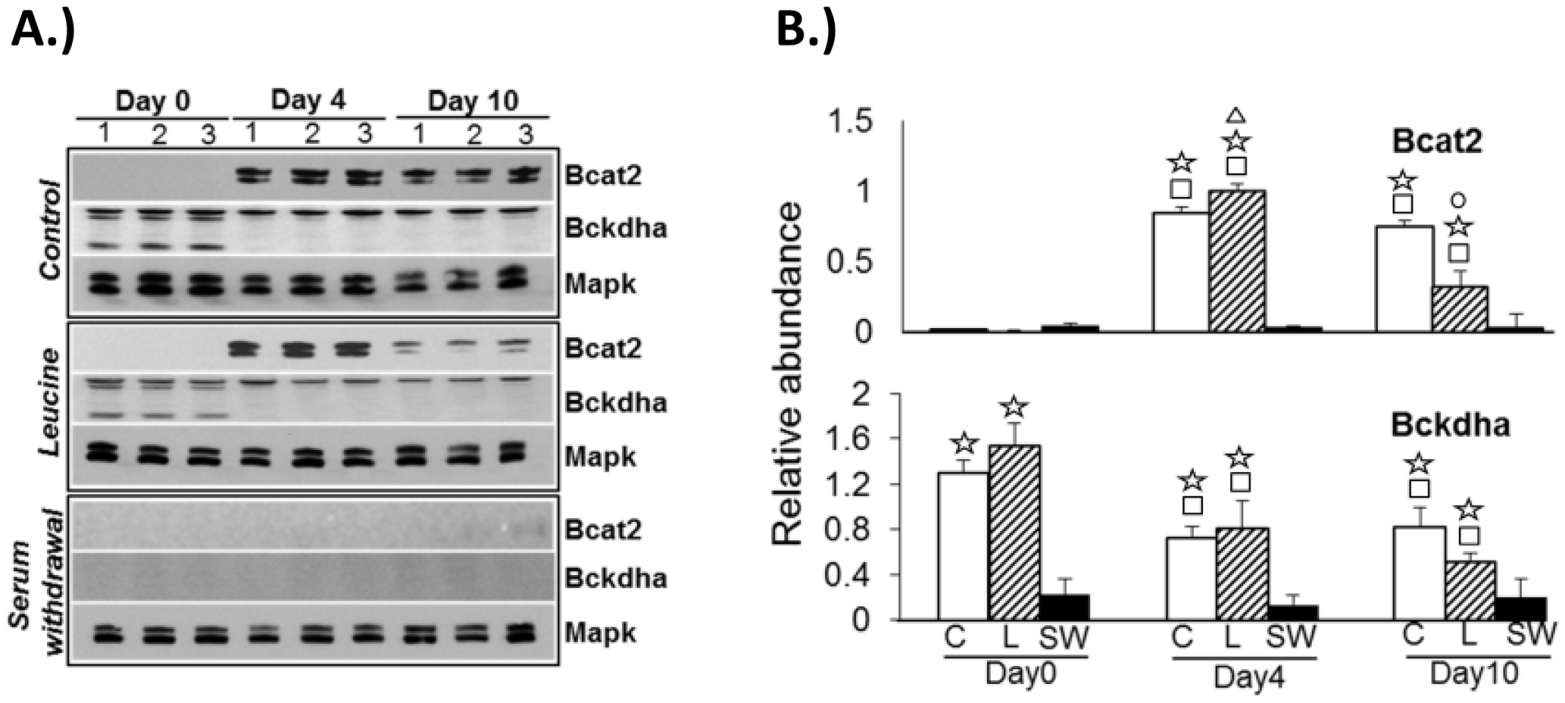
Western blot analysis of Bcat2 and Bckdha protein levels in treated cells over the course of adipocyte differentiation. **a.** Protein expression from three independent cell-culture experiments is shown for each gene. Treatments (*control, leucine or serum withdrawal*) are indicated at the left of each blot and the timepoints are indicated at the top. Mitogen activated protein kinase (Mapk) was used as loading control. **b.** Quantification of relative protein abundances by scanning densitometry on Western blots. Protein abundances are expressed relative to Mapk levels and average values from three independent cell-culture studies are shown. Significant changes in protein expression (p<0.05) are indicated as follows – comparison vs. Day 0, open squares; comparison against *serum withdrawal*, open star; comparison of Day 10 vs. Day 4, open circle; comparison of *leucine* vs. *control*, open triangle.

Bcat2 and Bckdha protein expression were analyzed via Western blots on replicate cell cultures for each treatment, using mitogen activated protein kinase (Mapk) as the loading control (**Figure 4**). Bcat2 expression was undetectable in the pre-adipocyte stage (Day 0) for both *control* and *leucine* treatments. A significant increase in expression was observed during early adipogenesis (Day 4) that persisted into the differentiated state (Day 10) for *control* samples (p<0.0001 for Day 4 and Day 10 vs. Day 0). Bcat2 protein induction showed greater sensitivity to *leucine* treatment at Day 4 but was significantly reduced on Day 10, compared to *control*. For *leucine* treatments, an even greater upregulation of Bcat2 protein levels was observed at Day 4 with significant reductions at Day 10 (p<0.0001 for Day 4 and Day 10 vs. Day 0; p<0.0001 for *leucine* Day 4 vs *control* Day 4 or *leucine* Day 10 vs. *control* Day 10). *Serum-withdrawal* led to barely detectable Bcat2 levels at all timepoints. A similar analysis for Bckdha showed the presence of 3 major bands in the Control Day 0 samples at 25, 47 and 50 kDa. The predicted molecular weight for murine Bckdha is 50 kDa, so the lower molecular weight bands could be degradation products (murine Bckdha is predicted to exist only as a single transcript). Consideration of the 50 kDa band showed the protein to be comparably expressed in the *control* and *leucine* treated samples across all timepoints. *Serum-withdrawal* again resulted in barely detectable Bckdha levels at all timepoints tested.

## Discussion

Several recent studies have pointed to interplay between adipose tissue, BCAA metabolism, and insulin signaling/glucose homeostasis. For example, adipose tissue specific overexpression of the glucose transporter *Glut4* was shown to result in a coordinate downregulation of BCAA metabolism genes selectively in epididymal adipose tissue, whereas the converse was true for *Glut4* knockout mice [17]. Transplantation of adipose tissue from wild-type mice into *Bcat2*-/- mice was further found to reduce post-absorptive plasma BCAA levels by 46%, demonstrating that adipose tissue can be a significant source of BCAA catabolism *in vivo* [17]. Other studies in humans and animal models have also largely established a negative correlation between obesity (and insulin resistance) and BCAA metabolism. For example, metabolomic analysis of blood has consistently demonstrated increases in circulating BCAA and their metabolites in obesity, suggesting altered flux through the BCAA catabolic pathway [18], [20], [40]. Conversely, large decreases in circulating BCAA levels have been observed in response to bariatric surgery in humans [41] along with increased expression in *Bcat2* and *BCKDHA* in subcutaneous and omental fat, suggesting a possible role of adipose tissue in regulating BCAA homeostasis following weight-loss [16]. These and other studies [18] have suggested that higher tissue and blood concentrations of BCAA may cause or exacerbate insulin resistance in obesity through leucine mediated activation of mTOR. However, the direction of causality and the specificity of the BCAA effect have been questioned in some other studies. For example, the observed drop in BCAA levels in bariatric surgery patients and the concurrent improvements in insulin sensitivity is equally plausible if inhibition of BCAA is a consequence and not a cause of insulin resistance. In other studies, the reduction in insulin-stimulated glucose uptake in response to BCAA or leucine was also mimicked by other amino acids such as methionine, hisitidine, threonine and tyrosine [42]. Whether the level of increases in blood BCAA levels are adequate to activate mTOR has also not been conclusively established and even when mTOR activation is observed (for example by doubling the intake of leucine in high-fat fed mice), metabolic and inflammation-related phenotypes are often either improved or normalized [34], [35]. Finally, the *Bcat2* knockout mice actually display improvements in insulin resistance rather than its exacerbation [43]. Therefore, the evidence for a contributory effect of elevated BCAA levels to obesity-associated insulin resistance is at best, equivocal [25].

Given this context, our study provides additional insights into the regulation of BCAA metabolism in adipocytes. We selected two opposing treatment conditions to exemplify nutrient excess (leucine supplementation) or nutrient restriction (serum withdrawal). We selected leucine as the BCAA of choice for supplementation studies, given its nutritional relevance as a major component of dietary proteins. Serum withdrawal was selected to mimic reduced nutrient supply such as may be encountered during starvation.

A general trend observed for the majority of the genes tested in this study was an increase in relative expression during early adipogenesis (Day 4) compared to the pre-differentiation (Day 0) or late differentiation (Day 10) stages. This trend was statistically significant for all genes (p<0.05) and observed in the *control* samples, suggesting that the behavior was intrinsic to adipocyte differentiation. The only two exceptions were for *Hmgcs1* and *Mcee* with the former showing a reduction, and the latter displaying no changes in their respective expression levels during adipocyte differentiation. Western blot analysis showed the temporal pattern of Bcat2 protein expression to be generally consistent with that of its message for the *control* samples, (with the exception of preadipocytes where there was no detectable protein expression). Bckdha protein expression was more discordant with its gene expression in that the highest level of protein was observed in the preadipocyte samples with lower levels in the Day 4 or Day 10 samples.

Exposure of cells to increased levels of leucine induced a statistically significant increase in *Bcat2* and *Bckdha* gene expression (compared to *control*) when treatments were initiated early (Day 4) or late (Day 10) (p<0.05). The amount of exogenous leucine used in these studies was 62.5% of the basal leucine levels already present in the media (0.8 mM). As previously noted, this level of leucine increment is in the range of previous leucine overfeeding studies or post-absorptive increases in leucine levels in obese mice [16], [35]. Leucine treatment did not lead to significant changes in the expression of valine-metabolizing genes, suggesting specificity in the transcriptomic response. Although several genes involved in leucine and isoleucine metabolism displayed reduced expression in response to leucine at Day 0 (compared to controls), the changes were statistically significant only for the *Acaa1b* and *Auh* genes. The significance of this finding is currently unclear and will be better ascertained when metabolic flux data becomes available. Western blot analysis showed an overall similar pattern of expression for Bcat2 and Bckdha in the leucine-treated samples as the control samples, except for Day 10 where both Bcat2 and Bckdha levels were significantly lower in the leucine-treated group. This may reflect substrate-level inhibition of BCAA metabolism, reported previously in rat heart [44], rat hepatocytes [45] and L6 myotubes [46] where, in the presence of other oxidizable substrates such as fatty acids, BCAA catabolic flux is inhibited via Bckdha inactivation [25]. We speculate that a similar mechanism may be operative in differentiating and differentiated adipocytes where the availability of fatty acids and glucose reduces the need for BCAA catabolism to tricarboxylic cycle intermediates and the excess leucine may instead be channeled to directly regulate other biological processes (e.g. translation initiation and insulin signaling via the mammalian target of rapamycin [47]).

Another key finding from the study was a statistically significant (p<0.05) upregulation of expression of several genes under conditions of *serum withdrawal*. Although this result may appear to be counter-intuitive, serum withdrawal is known to elicit complex and unpredictable time-dependent effects leading to diametrically opposing responses even in the same cell type [48]. Since several BCAA metabolizing genes are also involved in the catabolism of fatty acids, we hypothesize that the observed coordinated upregulation could reflect an adaptive transcriptomic response of the cells to mobilize lipid oxidation in the absence of external nutrient supply [49], [50]. Exceptions to this starvation related induction were noted for *Bckdh*a and *Hmgcs1* in the differentiating (Day 4) and differentiated (Day 10) samples, where the message levels were reduced compared to the *control* or *leucine* treated samples. However, both Bcat2 and Bckdha protein levels were barelydetectable under serum-withdrawal conditions at all time-points tested, suggesting a lack of correlation between protein and message levels, at least for these two proteins. We postulate this loss of Bcat2 and Bckdha will effectively block BCAA catabolism in the serum-deprived state, possibly in an effort to conserve BCAAs for protein synthesis, as previously noted in starved rats [1].

To summarize, the main findings from the study are as follows. First, the available data points to a complex relationship between mRNA and protein levels for two central BCAA metabolism genes. Thus, whereas concordance was noted in the increased message and protein levels of Bcat2 and Bckdha during early (Day 4) differentiation compared to pre- or late differentiation, serum-withdrawal led to increases in message levels of most BCAA metabolism genes whereas the protein levels for Bcat2 and Bckdha were undetectable. Second, the absence of Bcat2 protein expression, even in the presence of high Bckdha levels, in the Day 0 samples suggests a lack of direct oxidative metabolism of BCAAs in the preadipocyte. In this regard, the situation is similar to the liver where Bcat2 is nearly absent whereas Bckdha activity is maintained at high levels, possibly to enable clearance of branched-chain keto acids from portal blood. The function of Bckdha in the preadipocyte is, however, less clear. The capacity for oxidative degradation of BCAA appears to increase during the course of early adipocyte differentiation (Day 4) and is again reduced at or near completion of differentiation (Day 10), perhaps as the cellular focus shifts from lipid catabolism to lipid storage.

We should point out that the exposure periods used in our study (leucine or serum withdrawal) were comparatively short (18 hrs), implying that the findings may be more relevant for acute, and not chronic, nutrient effects. However, this experimental design was necessary since chronic exposure of adipocytes to serum deprivation led to a significant loss of cell viability. Additionally, the process of adipocyte differentiation was conducted in the presence of insulin 4 days after the start of differentiation after which insulin was withheld. The change in insulin exposure could influence the temporal gene expression. However, since the same differentiation procedure was followed for all treatments, we expect that insulin-dependent transcriptomic changes to be similar and therefore not a major confounder in our analysis. In summary, this study provides the first step in accurately quantifying the BCAA pathway transcriptome and comparing RNA and protein levels of two key regulators of BCAA metabolism (Bcat2 and Bckdha). Future studies will further investigate the phosphorylation status of Bckdha as well as quantify the overall flux through the BCAA catabolic pathways during adipocyte differentiation under conditions of nutrient stress.

## Supporting Information

Figure S1 Relative expression of BCAA pathway genes in 3T3-L1 preadipocytes. Gene expression levels were ascertained in three independent 3T3-L1 cell cultures. For each culture, quantitative PCR assays were conducted in triplicate and the average detection threshold (C_t_) and the corresponding standard deviations were plotted for each gene.
(TIF)

Figure S2 Changes in BCAA gene expression in response to treatments and to adipocyte differentiation. Gene expression changes are represented as fold-changes compared to Day 0 for each treatment. Results were averaged over three independent cell-culture experiments. Rows from top to bottom represent the following gene categories – *top row*, genes common to the metabolism of all 3 BCAAs; *second from top row*, genes specific to valine metabolism; *third from top row*, genes specific to leucine metabolism; *bottom row*, genes specific to isoleucine metabolism. Symbols for each gene are indicated at the top of the relevant plots. Treatments are indicated as follows – *open square*, control; *open triangle*, leucine supplementation; *open circle*, serum-withdrawal.
(TIF)

Table S1 qPCR primer sequences for BCAA metabolism genes. Primers are named in the format of ‘*genename_species_orientation*’; *m* represents mouse whereas *f* and *r* stand for forward and reverse primers, respectively.
(PDF)

Table S2 Assessment of the quality of total RNA used in the study. Isolated RNA was analyzed via Agilent Bioanalyzer. (**a**) RNA quality as determined from absorption spectroscopy (260/280 nm absorbance ratio) and from RNA Integrity Number (RIN) estimates. (**b,c**) Electropherograms of representative total RNA isolated from 3T3-L1 cells subjected to different treatments.
(PDF)

Table S3 Measurement of qPCR amplification efficiency for BCAA metabolism genes. PCR amplification efficiency was determined for each gene by 2-fold serial dilutions of cDNA reverse transcribed from the RNA. Column 1, gene name; column 2, slope of the linear fit of PCR detection threshold (Ct) vs. target cDNA concentrations; column 3, correlation coefficient of the linear fit; column 4, estimated amplification of the qPCR.
(PDF)

Table S4 Two-way ANOVA analysis of changes in BCAA metabolizing gene expression in response to treatments. The main effects of time and treatment and their interactions were analyzed. There were 3 levels for each factor (*Treatment*: Control, Leucine and Serum-withdrawal; *Time*: Day 0, Day 4, Day 10). Treatment means were calculated based on target gene expression, normalized to GAPDH expression levels, and then multiplied by a scaling factor of 10,000.
(PDF)
