# Supplementary figures and images for: Effects of Leucine Supplementation and Serum Withdrawal on Branched-Chain Amino Acid Pathway Gene and Protein Expression in Mouse Adipocytes
Source: PLoS One. 2014 Jul 22;9(7):e102615. doi: 10.1371/journal.pone.0102615 (PMC4106850; doi:10.1371/journal.pone.0102615)

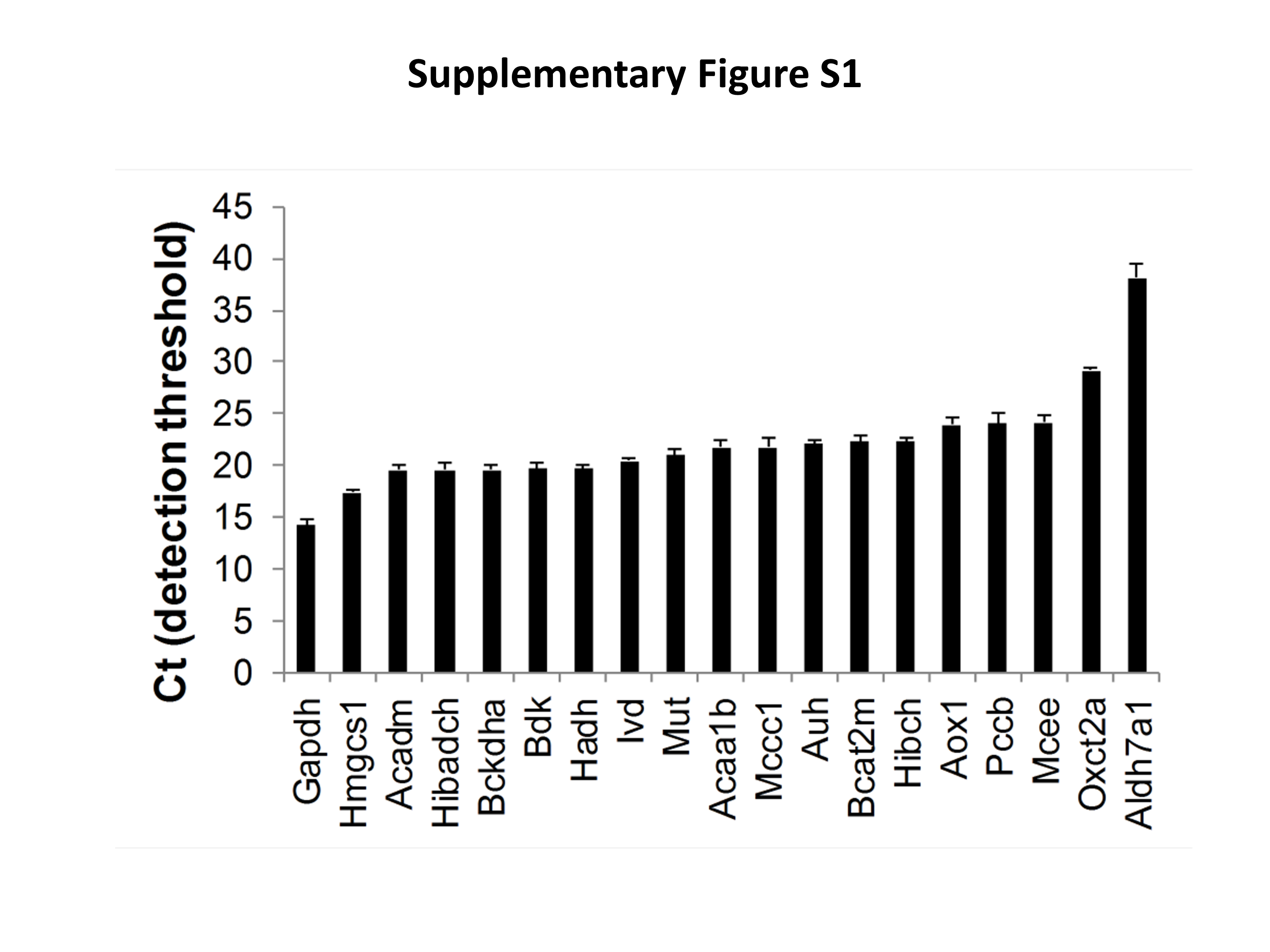

Supplement: Figure S1 — Relative expression of BCAA pathway genes in 3T3-L1 preadipocytes. Gene expression levels were ascertained in three independent 3T3-L1 cell cultures. For each culture, quantitative PCR assays were conducted in triplicate and the average detection threshold (Ct) and the corresponding standard deviations were plotted for each gene. (TIF) [file pone.0102615.s001.tif]

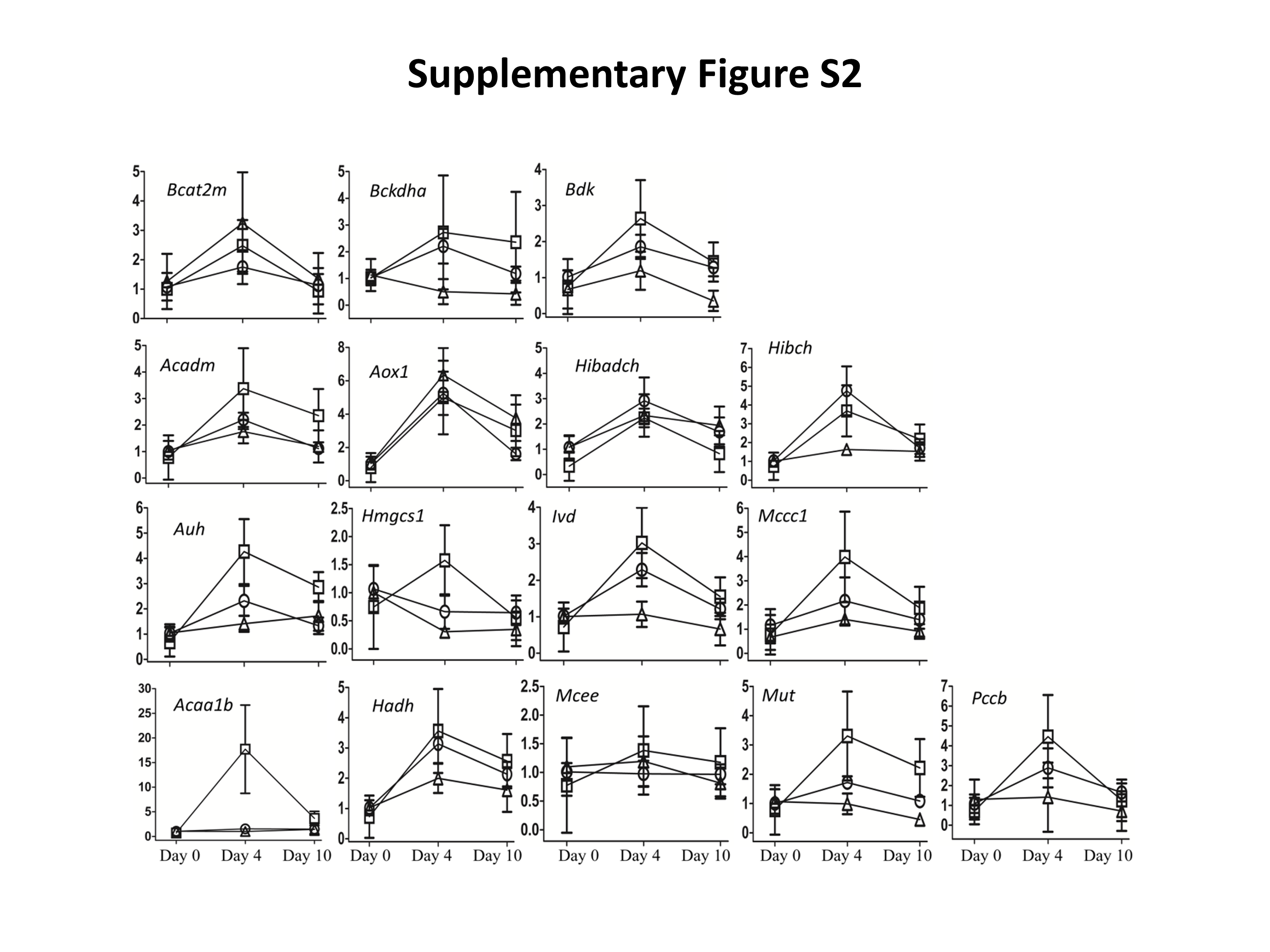

Supplement: Figure S2 — Changes in BCAA gene expression in response to treatments and to adipocyte differentiation. Gene expression changes are represented as fold-changes compared to Day 0 for each treatment. Results were averaged over three independent cell-culture experiments. Rows from top to bottom represent the following gene categories – top row, genes common to the metabolism of all 3 BCAAs; second from top row, genes specific to valine metabolism; third from top row, genes specific to leucine metabolism; bottom row, genes specific to isoleucine metabolism. Symbols for each gene are indicated at the top of the relevant plots. Treatments are indicated as follows – open square, control; open triangle, leucine supplementation; open circle, serum-withdrawal. (TIF) [file pone.0102615.s002.tif]
