# Supplementary material for: Effects of Leucine Supplementation and Serum Withdrawal on Branched-Chain Amino Acid Pathway Gene and Protein Expression in Mouse Adipocytes
Source: PLoS One. 2014 Jul 22;9(7):e102615. doi: 10.1371/journal.pone.0102615 (PMC4106850; doi:10.1371/journal.pone.0102615)
Supplement: Table S1 — qPCR primer sequences for BCAA metabolism genes. Primers are named in the format of ‘genename_species_orientation’; m represents mouse whereas f and r stand for forward and reverse primers, respectively. (PDF) [file pone.0102615.s003.pdf]

Supplementary Table S1: Sequence of qPCR primers for BCAA metabolizing genes used in the study. Primers are named as *'genename_species_orientation'*; *m* indicates *mouse* whereas *f* and *r* represent *forward* and *reverse* primers respectively.

Gene Symbol	SequenceName	Sequence
Acaa1b	Acaa1b_m_f	GAGACTGCCTGATTCCTATGG
	Acaa1b_m_r	GCACAATCTCAGCATGGAAG
Acadm	Acadm_m_f	TATCCCCGTCGCCCCGGAAT
	Acadm_m_r	GGCCAAGGCCACCGCAACT
Aldh7a1	Aldh7a1_m_f	GGGCGAGCGTCACCATCACA
	Aldh7a1_m_r	GGCTGGGACCTTCCTTGGGGA
Aox1	Aox1_m_f	CTGTACCCTCAACTTACTGTCC
	Aox1_m_r	ACACAAACTCCCACTTCCTG
Auh	Auh_m_f	CGACTTAGTAACAGGGTTAGCC
	Auh_m_r	CTCCTATTCTCCCTTGTAGCG
Bcat2m	Bcat2_m_f	TGGAGTGGAATAACAAGGCTG
	Bcat2_m_r	GTCTCCACCTTTGTATGCTTTC
Bckdha	Bckdha_m_f	CAGATGCCTGTTCACTACGG
	Bckdha_m_r	CCCTCGCCAAAGTAACAGATC
Bdk	BDK_m_f	CGTCTGGCCTCGCGATGGAA
	BDK_m_r	TGAGCGAGCCCGGAGTGACA
GAPDH	GAPDH_m_f	AGTGCCAGCCTCGTCCCGTA
	GAPDH_m_r	GCCACTGCAAATGGCAGCCC
Hadh	Hadh_m_f	AGGCTACACGAGCGAGGCGA
	Hadh_m_r	ACGGACCCATGGGATACCCAGC
Hibadch	Hibadch_m_f	CCCCTGTCTCCGGTGGTGTAGG
	Hibadch_m_r	GCCCCGTTCCAACAGCTCCAC
Hibch	Hibch_m_f	CTGAGTCTGAATATGATCCGGC
	Hibch_m_r	TGAGAGTGCTTTGATGTCCC
Hmgcs1	Hmgcs1_m_f	TTTGACCGAGGGCTCCGTGG
	Hmgcs1_m_r	TCCAGGGCGCTGAGGTAGCA
Ivd	Ivd_2_m_f	GGCATCACAGCCCCCGTTCA
	Ivd_2_m_r	AGCACCGTAGCTGAGCCCCA
Mccc1	Mccc1_m_f	AGGAAAAGGCATGAGGATCG
	Mccc1_m_r	AACACCTGGACTTCTACATGC
Mcee	Mcee_m_f	AGGAATGCATCACGTCTGC
	Mcee_m_r	CACAGTCTTTGGGATGGAGG
Mut	Mut_m_f	AGGCCCTGGACCATCCGTCA
	Mut_m_r	CAACCCCTGCTGACCAGCCTT
Oxct2a	Oxct2a_m_f	GGTAGATGACATCAAGGCCAC
	Oxct2a_m_r	TGCTAAGGATGTCCAGTTGTC
Pccb	Pccb_m_f	TGATGCTCTGTGTAACCTGC
	Pccb_m_r	TGTAGGCTTTGCTTGACTCC
