# Supplementary material for: Effects of Leucine Supplementation and Serum Withdrawal on Branched-Chain Amino Acid Pathway Gene and Protein Expression in Mouse Adipocytes
Source: PLoS One. 2014 Jul 22;9(7):e102615. doi: 10.1371/journal.pone.0102615 (PMC4106850; doi:10.1371/journal.pone.0102615)

Supplementary Table S2: Assessing the quality of total RNA. Isolated total RNA was subjected to analysis on an Agilent Bioanalyzer. **(a)** Quality of the RNA was determined by the 260/280 ratio and the RIN estimates. **(b,c)**The electropherograms for representative total RNA isolated from 3T3-L1 cells undergoing different treatments are shown.

a.

Sample ID	260/280	RIN #
Control, Day 0	2.09	9.9
Control, Day 4	2.19	9.6
Control, Day 10	2	9.8
Serum starved, Day 0	2.01	9.6
Serum starved, Day 4	2.08	9.7
Serum starved, Day 10	2.11	9.2
Leucine, Day 0	2.08	10
Leucine, Day 4	2.07	9.8
Leucine, Day 10	2.09	9.9

b.

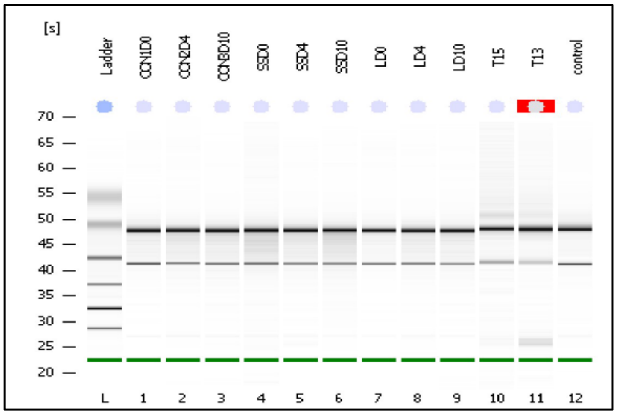

c.

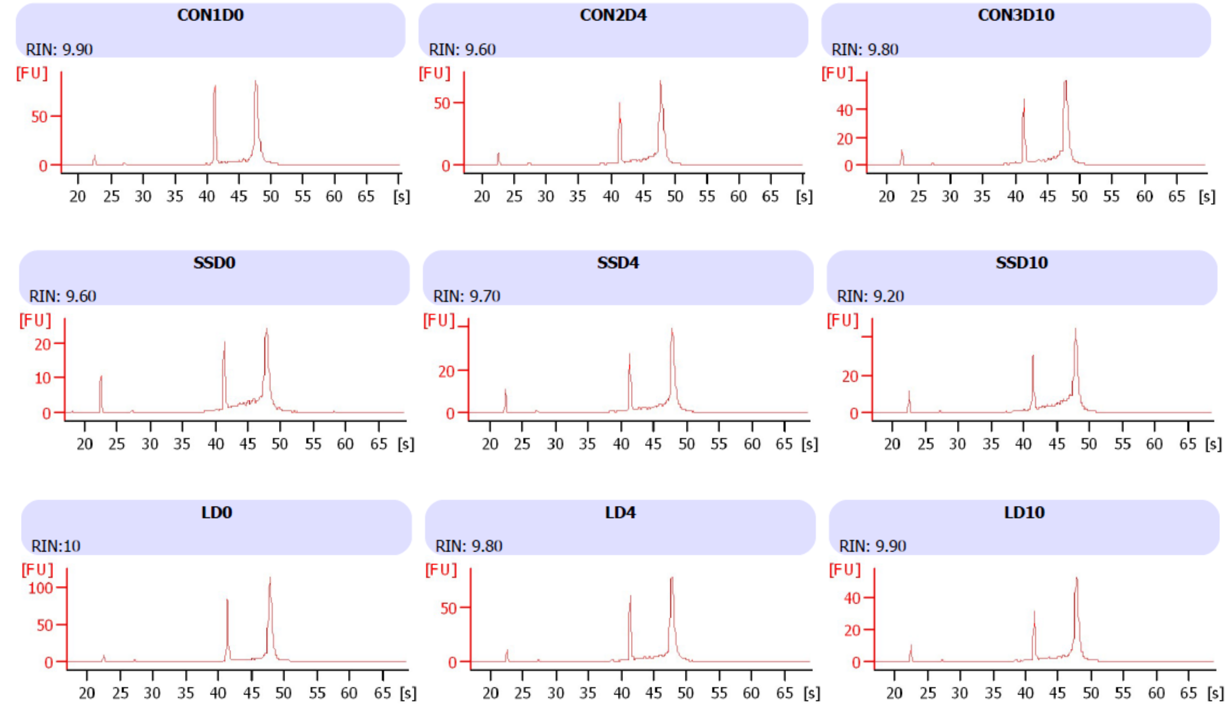

Supplement: Table S2 — Assessment of the quality of total RNA used in the study. Isolated RNA was analyzed via Agilent Bioanalyzer. (a) RNA quality as determined from absorption spectroscopy (260/280 nm absorbance ratio) and from RNA Integrity Number (RIN) estimates. (b,c) Electropherograms of representative total RNA isolated from 3T3-L1 cells subjected to different treatments. (PDF) [file pone.0102615.s004.pdf]
