# Supplementary material for: Effects of Leucine Supplementation and Serum Withdrawal on Branched-Chain Amino Acid Pathway Gene and Protein Expression in Mouse Adipocytes
Source: PLoS One. 2014 Jul 22;9(7):e102615. doi: 10.1371/journal.pone.0102615 (PMC4106850; doi:10.1371/journal.pone.0102615)
Supplement: Table S3 — Measurement of qPCR amplification efficiency for BCAA metabolism genes. PCR amplification efficiency was determined for each gene by 2-fold serial dilutions of cDNA reverse transcribed from the RNA. Column 1, gene name; column 2, slope of the linear fit of PCR detection threshold (Ct) vs. target cDNA concentrations; column 3, correlation coefficient of the linear fit; column 4, estimated amplification of the qPCR. (PDF) [file pone.0102615.s005.pdf]

Supplementary Table S3: QPCR amplification efficiency for BCAA metabolism genes. PCR amplification efficiency was determined for each gene by serial dilutions of target cDNA, described in the text. *Column 1*, gene name; *column 2*, slope of linear fit of Ct values vs. target concentrations; *column 3*, correlation coefficient of linear fit; *column 4*, calculated amplification efficiency of qPCR.

Gene	Slope	R2	Amplification Efficiency (%)
GAPDH	-3.1997	0.999	105.37
HMGCS1	-3.3495	0.992	98.84
BCAT2	-3.3514	0.976	98.87
BCKDA	-3.4179	0.998	96.15
ACADM1	-3.4329	0.980	95.57
HADH	-3.2917	0.996	101.35
IVD	-3.0993	0.949	110.21
HIBADCH	-3.3769	0.998	97.80
BDK	-3.1172	0.993	109.32
HIBCH1	-3.1267	0.992	108.85
MCCC1	-3.2010	0.977	105.31
ACAA1B	-3.1046	0.984	109.92
MUT	-3.1647	0.982	107.01
AUH	-3.4425	0.978	95.22
PCCB	-3.3561	0.998	98.60
AOX	-2.9892	0.988	101.35
MCEE	-3.4425	0.977	108.80
