# Supplementary material for: Effects of Leucine Supplementation and Serum Withdrawal on Branched-Chain Amino Acid Pathway Gene and Protein Expression in Mouse Adipocytes
Source: PLoS One. 2014 Jul 22;9(7):e102615. doi: 10.1371/journal.pone.0102615 (PMC4106850; doi:10.1371/journal.pone.0102615)
Supplement: Table S4 — Two-way ANOVA analysis of changes in BCAA metabolizing gene expression in response to treatments. The main effects of time and treatment and their interactions were analyzed. There were 3 levels for each factor (Treatment: Control, Leucine and Serum-withdrawal; Time: Day 0, Day 4, Day 10). Treatment means were calculated based on target gene expression, normalized to GAPDH expression levels, and then multiplied by a scaling factor of 10,000. (PDF) [file pone.0102615.s006.pdf]

**Supplementary Table S4: Two way ANOVA analysis of BCAA metabolism gene expression.** The main effects of time and treatment and their interactions were analyzed. There were 3 levels for each factor (Treatment: *Control*, *Leucine*, *Serum withdrawal*; Time: *Day0*, *Day4*, *Day 10*). Treatment means were calculated based on target gene expression normalized to GAPDH levels and then multiplied by a factor of 10,000.

| Genes | Treatment Mean(SEM) | | | ANOVA p-values | | | |
|---|---|---|---|---|---|---|---|
| | | | | Full Model | Treatment | Time | Treatment * Time |
| ACAA1B | 70.72 (12.20) | 52.82 (13.24) | 152.53 (14.15) | 0.0007 | <.0001 | 0.0126 | 0.0404 |
| ACADM1 | 317.42 (48.84) | 283.73 (52.75) | 771.23 (48.84) | <.0001 | <.0001 | 0.0012 | 0.7397 |
| AOX1 | 32.76 (3.57) | 40.67 (3.85) | 75.92 (3.57) | <.0001 | <.0001 | <.0001 | 0.0007 |
| AUH2 | 72.17 (8.57) | 53.17 (9.26) | 161.41 (8.57) | <.0001 | <.0001 | 0.0015 | 0.1045 |
| BCAT2 | 52.37 (9.33) | 102.51 (10.08) | 93.70 (9.33) | 0.0004 | 0.0045 | 0.0001 | 0.186 |
| BCKDA | 312.70 (38.03) | 505.56 (43.91) | 436.86 (43.91) | 0.0004 | 0.0033 | 0.0072 | 0.0006 |
| BDK | 323.57 (50.33) | 372.23 (54.26) | 625.20 (58.11) | 0.0057 | 0.0063 | 0.0056 | 0.3151 |
| HADH | 524.89 (66.18) | 551.98 (71.40) | 910.51 (66.18) | 0.0006 | 0.0008 | 0.0001 | 0.9986 |
| HIBADCH1 | 441.53 (50.17) | 442.54 (67.93) | 884.34 (50.17) | <.0001 | <.0001 | 0.0002 | 0.4248 |
| HIBCH2 | 99.79 (9.73) | 91.84 (10.51) | 168.88 (9.73) | <.0001 | <.0001 | <.0001 | 0.0606 |
| HMGCS | 1013.50 (103.09) | 510.37 (111.35) | 915.42 (103.09) | 0.0009 | 0.0107 | 0.0019 | 0.0312 |
| IVD2 | 228.14 (23.82) | 190.18 (25.73) | 265.78 (23.82) | 0.0078 | 0.0791 | 0.001 | 0.2302 |
| MCCC1 | 95.55 (8.32) | 83.19 (8.99) | 152.97 (8.99) | <.0001 | <.0001 | <.0001 | 0.6392 |
| MCEE2 | 14.23 (1.83) | 10.22 (1.98) | 28.98 (1.83) | 0.0002 | <.0001 | 0.2904 | 0.4904 |
| MUT | 144.50 (16.05) | 124.86 (17.34) | 232.28 (16.05) | 0.0003 | 0.0003 | 0.0027 | 0.0128 |
| OXCT2A | 1.56 (0.23) | 0.36 (0.25) | 1.24 (0.27) | 0.0001 | 0.0089 | 0.0018 | 0.0004 |
| PCCB2 | 24.70 (2.64) | 14.04 (2.85) | 20.06 (3.05) | 0.0055 | 0.0206 | 0.0423 | 0.0185 |
